# The answer is 17 years, what is the question: understanding time lags in translational research

**DOI:** 10.1258/jrsm.2011.110180

**Published:** 2011-12

**Authors:** Zoë Slote Morris, Steven Wooding, Jonathan Grant

**Affiliations:** 1 Institute of Public Health, University of Cambridge, Cambridge CB2 0SR, UK; 2 RAND Europe, Cambridge CB4 1YG, UK

## Abstract

This study aimed to review the literature describing and quantifying time lags in the health research translation process. Papers were included in the review if they quantified time lags in the development of health interventions. The study identified 23 papers. Few were comparable as different studies use different measures, of different things, at different time points. We concluded that the current state of knowledge of time lags is of limited use to those responsible for R&D and knowledge transfer who face difficulties in knowing what they should or can do to reduce time lags. This effectively ‘blindfolds’ investment decisions and risks wasting effort. The study concludes that understanding lags first requires agreeing models, definitions and measures, which can be applied in practice. A second task would be to develop a process by which to gather these data.

## Introduction

Timely realization of the benefits of expensive medical research is an international concern attracting considerable policy effort around ‘translation’.^1,2^ Policy interventions to improve translation respond to a vast empirical literature on the difficulties of getting research across research phases and into practice.^3–11^

Both literature and policy tend to assume that speedy translation of research into practice is a good thing. Delays are seen as a waste of scarce resources and a sacrifice of potential patient benefit.^12^ Although some lag will be necessary to ensure the safety and efficacy of new interventions or advances, in essence we should aim to optimize lags. One recent study (of which JG and SW were co-authors) estimating the economic benefit of cardiovascular disease (CVD) research in the UK between 1975 and 2005, found an internal rate of return (IRR) of CVD research of 39%.^13^ In other words, a £1.00 investment in public/charitable CVD research produced a stream of benefits equivalent to earning £0.39 per year in perpetuity. Of this, 9% was attributable to the benefit from health improvements, which is the focus of this paper. (The remaining 30% arise from ‘spillovers’ benefiting the wider economy.) This level of benefit was calculated using an estimated lag of 17 years. Varying the lag time from 10 to 25 years produced rates of return of 13% and 6%, respectively, illustrating that shortening the lag between bench and bedside improves the overall benefit of cardiovascular research. What is notable is that all the above calculations depended upon an estimated time lag; estimated because, despite longstanding concerns about them,^14^ time lags in health research are little understood.

It is frequently stated that it takes an average of 17 years for research evidence to reach clinical practice.^1,3,15^ Balas and Bohen,^16^ Grant^17^ and Wratschko^18^ all estimated a time lag of 17 years measuring different points of the process. Such convergence around an ‘average’ time lag of 17 years hides complexities that are relevant to policy and practice which would benefit from greater understanding.^13^

Despite longstanding concerns about delays in getting research into practice, the literature on time lags seems surprisingly under-developed. To help address this gap, this paper aims to synthesize existing knowledge and to offer a conceptual model that can be used to standardize measurement and thus help to quantify lags in future. This would allow efforts to reduce lags to be focused on areas of particular concern or value, or on areas where interventions might be expected to have best effect. It would also provide the potential for evaluating the cost-effectiveness of translation interventions if their impact on lags can be measured. The aim was to overlay empirical lag data onto the conceptual model of translational research to provide an overview of estimated time lags and where they occur. The first part of the paper explores conceptual models of the translation pipeline in order to provide context. The second part of the paper presents a review of the literature on time lags to present current estimates and issues. This leads to a discussion on the current state of understanding about time lags and considers the implications for future practice and policy.

## Methods

For the first part of the study we identified literature that described conceptual models of translation. Our search was not intended to be exhaustive, but included key policy documents and searches of Google Scholar, Web of Science, PubMed and EBSCO. Key words used to retrieve relevant studies included ‘valley of death’, ‘bench to bedside’, ‘translational research’ and ‘commercialisation’. In general, ‘grey’ literature was not included in the search, but the HERG study^19^ was included because of the authors' involvement in it. The models in the literature found by these methods were summarized into a simple conceptual model.

For the second part of the study we reviewed the literature on time lags in health research. We used the same methods and literature as for the first part but included additional search terms such as ‘time lag’ or ‘time-lag’, ‘delays’, ‘time factors’ (PubMed MESH term) and ‘publication bias’. We found a formal search yielded few relevant papers so combined a number of approaches to increase our confidence that relevant papers had been identified. We undertook backward and forward citation tracking to identify related work and used searches within targeted journals – e.g. *Scientometrics* and *Journal of Translational Medicine*. To analyse the lag data, we used a data extraction template with the following fields: start and end dates for measurement period, range, mean, median, dates used, topic, country of study. In addition, the start point and endpoint of the time lag measured in each study were mapped onto specific stages in the conceptual model developed in the first part of the study.

## Findings

### Conceptualising translational research

Understanding time lags requires a conceptual model of how research in science is converted to patient benefit so that the durations of activities and waits can be measured. This process of conversion of basic science to patient benefit is often called ‘translation’.^1,2,18,20–22^ Woolf has argued that ‘translation research means different things to different people’^23^ and this is reflected in the various models and definitions found in the literature. However, as translational research also ‘seems important to almost everyone’^23^ there would seem to be benefit in trying to unify models and definitions.

**Figure 1:**
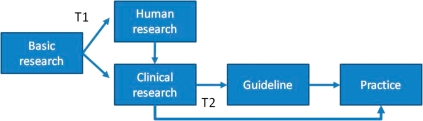
A conceptual model of the journey of health (biomedical) research from research into benefit, as derived from the literature

We have attempted to synthesize these models to identify key features of the translation process and to offer a tentative unified model. This was intended to help stakeholders agree a model which could be used to support future data gathering and better guide policy-making. We recognized that drug development, public health, devices and broader aspects of healthcare practice will vary in nature. The translation process is summarized briefly in Figure 1. Clearly this model can be critiqued for being linear and we acknowledge the considerable literature that challenges this notion and accept that research translation is a messy, iterative and complex process (see Balaconi *et al.* for a good review of the liner models critiques and their partial rebuttal^24^). At the same time, we would argue that for the purposes of understanding and conceptualising time lags the model is appropriate in showing common steps found in the literature.

‘Translational research’ is typically separated into two phases of research. Type 1 translation, also somewhat confusingly called ‘bench to bedside’, refers to the conversion of knowledge from basic science research into a potential clinical product for testing on human subjects. Type 2 translation, ‘research into practice’, tends to refer to the process of converting promising interventions in clinical research into healthcare practice (thus is closer to the notion of the ‘bedside’).^2,20,21,25,26^ Each phase of translational research is associated with a set of research activities which contribute to lags.^27^ These include processes around grant awards, ethical approvals, publication, phase I, II, III trials, approvals for drugs, post-marketing testing, guideline preparation and so forth. Some of these activities are repeated in different phases – grants and publications most particularly. Each activity involves a lag, either because the effort required for carrying out the task or as a result of non-value adding waits. The activities are used as ‘markers’ in studies of lags.

Conceptual models typically include ‘translational gaps’, which describe the movement from one phase of research to another. Each of these is also associated with delays, although precisely what and where these gaps are, and how long they are, is again not consistent in the literature. Policy measures to expedite the translation process typically focus on these gaps.

### Estimating time lags in the translation process

Table 1 shows a summary of estimates derived from empirical studies of lags.

**Table 1:** Summary of studies of time lags in health research

Author	Context	Start of time lag	End of time lag	Time lag (years)				Dates	Country	Notes
				Lower range	Median	Mean	Higher Range			
Antman (1992)^38^	Treatment for myocardial infarction	Publication of clinical trial	Guideline/ recommendation	6			13	1966–1992	US	
Altman (1994)^46^	Statistical techniques	First publication	Highly cited	4			6			
Balas and Bohen (2000)^16^	Various	‘Original research’	Implementation			17		1968–1997	International	Calculated from adding a number of studies together
Cockburn and Henderson (1996)^53^	Drugs	Date of enabling scientific research	Date to market	11		28	67	‘Narrative histories’ of drug discoveries, 1970–1995	US	
Comanor and Scherer (1969)^55^	Drugs	Patent	New entities		3	3			US	
Comroe and Dripps (1976)^36^	‘Top ten clinical advances in cardiovascular and pulmonary medicine and surgery’ – ECG	Publication	Clinical advances				306	Key advances since 1945	US	
Contopoulos- loannidis (2008)^35^		Publication (First description)	First specific use	0			221	High citations in 1990–2004	International	Worked backwards from highly cited (over 1000 citations on WoS) to the first description; interquartile range
Contopoulos- loannidis (2008)^35^		Publication (First description)	Highly cited publication	14	24	24	44	High citations in 1990–2004	International	Worked backwards from highly cited (over 1000 citations on WoS) to the first description; interquartile range
Contopoulos- loannidis (2008)^35^		Publication (First description)	First human use	0			28	High citations in 1990–2004	International	Worked backwards from highly cited (over 1000 citations on WoS) to the first description; interquartile range
Decullier *et al.* (2005)^26^	Various	Ethics approval	Date for first publication					Ethical approval given in 1994; study conducted in 2000	France	Does not report for all papers, but only by direction of results; does not report ranges
DiMasi (1991)^56^	Not mentioned	Clinical testing	Submission to FDA			6.3			US drugs	
DiMasi (1991)^56^	Not mentioned	Clinical testing	Marketing approval			8.2			US drugs	
DiMasi (2003)^29^	R&D expenditure from 1980–1999	Clinical testing	Submission to FDA			6		1980–1999	US drugs	
DiMasi (2003)^29^	R&D expenditure from 1980–1999	Clinical testing	Marketing approval			7.5		1980–1999	US drugs	
Grant *et al.* (2000)^28^	Various	Publication	Guideline	0	8		49	1988–1995	UK guideline	Range estimated from Figure 1
Grant *et al.* (2003)^17^	Neonatal care	Publication	Most recent paper	13		17	21	1995–1999	UK	Estimated from graph
Harris *et al.* (2010)^40^	Cancer drugs	Abstract	Publication	0.4		0.75	1.6	2005–2007	UK	Results changed for abstract to full publications in 3 out of 3 cases
HERG *et al.* (2008)^13^	CVD	Publication	Guideline	9		13*	14	1975–2005	UK guideline	Range varied by topic; assume a three year lag in publication; and used the same study period
HERG *et al.* (2008)^13^	Mental health	Publication	Guideline	6		9	11	1975–2005	UK guideline	Range varied by topic; assume a three year lag in publication; and used the same study period
Ioannidis (1998)^31^	AIDS	Date of trial registration	Publication	3.9	5.5		7	Studies conducted between 1986 and 1996	US	Uses interquartile range
Ioannidis (1998)^31^	AIDS	Date of trial registration	Date of completion of study	2	2.6		3.8	Studies conducted between 1986 and 1996	US	Uses interquartile range
Ioannidis (1998)^31^	AIDS	Completion of study	First submission	0.7	1.4		2.3	Studies conducted between 1986 and 1996	US	Uses interquartile range
Ioannidis (1998)^31^	AIDS	First submission	Publication	0.6	0.8		1.4	Studies conducted between 1986 and 1996	US	Uses interquartile range; ‘negative studies suffer a substantial time lag. With some expectations, most of this lag is generated after a trial has been completed.’ (p. 284)
Mansfield (1991)^33^	Manufacturing products, including drugs	Academic research	Commercialization			7		1975–1985	US	Cites Gellman who calculated a lag of 7.2 year between (1953–1973)
Misakian and Biro (1998)^39^	Passive smoking	Funding began	Date of first publication describing health effects		3(+); 5–7 (–); 3 (incon)			Studies started between 1981 and 1995; study conducted 1995	US – study of funding bodies	Does not report for all papers, but only by direction of results; noted that tobacco-affiliated organizations did not respond to requests to take part in the study despite several requests
Pulido *et al.* (1994)^47^	Papers published in *Medicina Clínica*	Submission of paper	Publication	0.81		0.86	0.92	Looked at 12 articles in 5-year cycles, from 1962–1992; data for 1982	Spanish journal articles	Study is in Spanish; only seems to report data from two cycles (1982 and 1992)
Pulido *et al.* (1994)^47^	Papers published in *Medicina Clínica*	Submission of paper	Publication	0.32		0.81	0.56	Same study as above but, data for 1992	Spanish journal articles	Study is in Spanish; only seems to report data from two cycles (1982 and 1992)
Stern and Simes (1997)^8^	Quantitative studies submitted to Royal Prince Albert Hospital Ethics Committee	Ethical approval	Date of first publication	3.9 (+); 6.9 (– or inconc)			5.7 (+); ∞ (– or inconc)	Ethical approval given in 1979–1981; study conducted in 1992	Royal Prince Alfred Hospital Ethics Committee Applicants, Australia	Does not report for all papers, but only by direction of results
Stern and Simes (1997)^8^	Trials submitted to Royal Prince Albert Hospital Ethics Committee	Ethical approval	Date of first publication – trial data	3.7 (+); 7.0 (– or inconc)			5.7 (+); ∞ (– or inconc)	Ethical approval given in 1979–1981; study conducted in 1992	Royal Prince Alfred Hospital Ethics Committee Applicants, Australia	Does not report for all papers, but only by direction of results
Sternitzke (2010)^30^	‘Pharmacuetal products’; drugs approved by FDA	Chemical synthesis	FDA approval			11.5			US drugs	Sternitzke's estimates derive from a literature review
Wang-Gilam (2010)^25^	Cancer trials	Trial application	Enrolment	0.3			0.44	2001–2008	US; two centres	
Wratschko (2009)^18^	General pharma	Drug discovery	Commercialization	10		12	17		US book	Derived from LR Green (2005)

*The difference between this value and the 17 years cited in the introduction is that for this study the authors also took into account estimates between time of funding and publication and other studies (which are reviewed in this paper)

HERG = Health Economics Research Group at Brunel University

**Figure 2:**
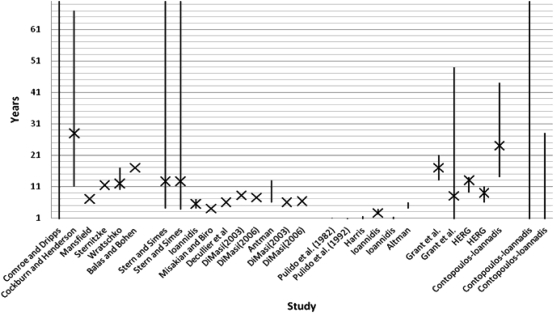
Chart showing the approximate range and average time lag reported in studies of time lags in health research. NB – HERG is the Health Economics Research Group at Brunel University

Figure 2 shows these time-lag estimates by research phase. Some additional ‘averaging’ has been necessary to provide single figures where ranges only were used in the original paper. The source data are presented in Appendix A (see http://jrsm.rsmjournals.com/content/104/12/510/suppl/DC1).

### Issues with measurement and estimation

As is shown in Table 1, studies of time lags in translation of research to practice often measure different points in the process. For example, Decullier *et al.*^28^ and Stern and Simes^29^ measure time between ethical approval and date of first publication; Grant *et al.*^30^ and HERG *et al.*^13^ look at publication to guideline; DiMasi calculated the length of time within and between phases in US drug development to calculate the costs associated with the phases.^31^ Sternitzke looked at commercialization of pharmaceutical innovations from ‘chemical synthesis’ to FDA approval.^32^ Ioannidis attempted to estimate the time lag between date of trial registration and several milestones to publication.^33^ Grant *et al.*, Mansfield and Comroe and Dripps work backwards from practice to publication.^17,34–36^

Not surprisingly given they are measuring different lags, Figure 2 helps show that data are generally sparse and estimates vary.^37^ Some studies report longer lags for publication to guideline^17,38^ than others do for development to commercialization.^18,32,35^ Table 1 also points to two substantive gaps in knowledge: the time lag involved in and between discovery and development (T1), and the time lag between publication to practice. Only one study has ‘implementation’ into practice as its endpoint.

Measurement and reporting is often poor. For example, Decullier *et al.* report ‘mean’ lags,^28^ and Dwan, in reporting Decullier *et al.*, in their review refer to ‘median’ lags.^36^ Neither reports distributions. Ranges – or even interquartile ranges as large as 221 years^38^ – are seldom reported. Furthermore, where it was possible, further investigation of the average revealed wide variation; variation which is not highlighted or discussed in the papers. For example, Hopewell *et al.* in their review of publication bias conclude that clinical trials with null or negative results ‘on average’ took ‘just over a year longer to be published than those with positive results’.^39^ This average is associated with a range of six to eight years for studies with a negative or null result, compared with four to five years for those with positive results. Comparing the slowest negative publication with the fastest positive publication makes a potential difference of four years – half of the maximum lag.

Some studies aggregate data from earlier studies without critical reflection or recognition of this.^16,25^ For example, Balas and Bohen calculate an average of 17 years from original research to practice formed from adding together a number of single studies of different phases including one that estimates a lag of 6–13 years.^16^ Accounting for this changes their estimate of the time lag between journal submission to use in practice from between 17 years to 23 years.

Not surprisingly, studies also show variation in time lags by domain^38^ and even intervention within a single domain. For example, examining research relating to advances in neonatal care, Grant *et al.* traced research papers back through four ‘generations’ of publication. They found ‘the overall time between generations 1 to 4 ranges from 13 years (for artificial surfactant) to 21 years (for parenteral nutrition). The other three advances took 17 years to develop through four generations of citations’. Atman *et al.*'s study of treatment for myocardial infarction yielded similar results: it took six years for a review of evidence supporting the use of thrombolytic drugs to result in a standard recommendation, whereas prophylactic lidocaine was used widely in practice for 25 years based on no evidence of effectiveness.^40^

Content also appears to influence time lags. A common theme found in the literature concerns publication bias, and their implications for judging effectiveness.^28,29,33,37–39,41–47^ Altman looked at citations of new statistical techniques applied to health and found that it took 4–6 years for a paper to receive 25 citations if the technique was new. An ‘expository article’ could achieve 500 citations over the same period.^48^ Contopoulos-Ioannidis found different publication trajectories for different types of invention.^38^

Studies also show that time lags are not stable over time. For example, Pulido noted a difference of 0.9 years or 0.3 years from acceptance to publication in 1992 and 1982, respectively.^49^ DiMasi reported a slight shortening of the approval process between 1991 and 2003.^31^ Tsuiji and Tsutani reported reduced lags in the drug approval process in Japan following a change in policy to try and expedite it.^50^

Single papers raise issues that are not generally discussed but do seem relevant to measuring time lags from publications in particular. These issues include ‘generations’ of research^19^ and overlaps in research publications.^19,33^ For example, Ducullier, of the 649 studies they included, five years later 59% had published research findings but most (84%) had more than one paper from the same study.^28^

## Discussion

This paper aimed to synthesize existing knowledge to offer a conceptual model that can be used to standardize measurement and thus help to quantify lags in future. The strengths of the study are that, to our knowledge, this provides the first attempt to review lags comprehensively, both in terms of using multiple approaches to find studies, but also in attempting to quantify time lags along the translation continuum. The review exposed a number of weaknesses in the literature and gaps in knowledge, which are not often discussed. Despite our attempts to be comprehensive, however, we are aware that studies of time lags in health research are widely distributed and not easily identified using formal literature searches and we may have failed to capture relevant studies. We struggled to find research quantifying lags in basic research and the first translation gap in particular.

Our aim to understand lags has been limited by the weaknesses of existing data. Limitations of the literature examined include the use of proxy measures. Much of the literature on lags focuses on dissemination and publication in peer-review journals in particular as these are the most measureable. If there are significant lags in, say, the grant or ethics process, this is less likely to be reflected in current total lag estimations. Moreover, the variation in choice of proxy measures means that studies are almost never measuring the same thing, making valid aggregation and generalization difficult.

There is a clear trend in the literature to seek a single answer to a single question through the calculation of an average. The variation found in the literature suggests that this is not possible (or even desirable), and variation matters. Moreover, many of the published ‘averages’ are derived from adding an empirically derived mean duration for one section of journey from one point in time, in one topic, and adding it to other parts without reflection. Thus any poor estimates are transferred forward into later analysis, and also hide a complexity which is highly relevant to research policy.

There also appears to be a mismatch between conceptual models of the translation process, and the measuring of lags. For example, the gap between guideline publication and translation into actual practice is often ignored, suggesting an under-estimation of the time lags in some cases. On the other hand, interventions may come into use before guidelines outlining them have been published – suggesting an over-estimation of time lags in other cases.

Using different endpoints, different domains and different approaches, Balas and Bohen^16^ and Grant *et al.*^30^ both estimate the time lag in health research being 17 years. Wratschko also suggested 17 years as the highest limit for the time taken from drug discovery to commercialization.^18^ It is surprising that 17 is the answer to several related but differing questions. Is this coincidence or not? One possible reason for the convergence is the difficulty of measuring longer lags – because of limitations of citation indexes, other records and recollections – which provides a ceiling to such estimates and leads to a convergence of average lags.

While not able to adequately quantify time lags in health research, this study provides lessons for future research policy and practice. Concerns about lags are not new^14^ but are unresolved. Based on the review, and our own work on lags,^13,17,19,30,51^ we would argue that an essential step to being able to quantify time lags, and thereby make improvements, requires stakeholders to agree definitions, key stages and measures. It also perhaps requires stakeholders to develop a more nuanced understanding of when time lags are good or bad, linked to policy choices around ethics and governance for example,^52^ or reflect workforce issues.^52,53^ Indeed, a recent paper by Trochmin *et al.*^54^ proposes a ‘process maker model’ whereby they identify a set of operational and measureable markers along a generalized pathway like that illustrated in Figure 1. It seems to us that this provides an excellent framework to support future data gathering and analysis and thus provide a more informed base from which to develop policy to address time lags.

Currently much of the complexity, and therefore the potential for improvement, are hidden in this preference for ‘averages’. No attention is given to understanding distributions and variations. This effectively ‘blindfolds’ investment decisions and risks wasting efforts to reduce lags. As noted in the introduction, some lags are necessary to ensure the safety and efficacy of implementing new research into practice. The discussion in the literature fails to consider what is necessary or desirable, tending to assume that all lags are unwelcome. A key question for policy is to identify which lags are beneficial and which are unnecessary, but to answer this question it is necessary to have an accurate and comparable estimate of the lags.

## Conclusion

Translating scientific discoveries into patient benefit more quickly is a policy priority of many health research systems. Despite their policy salience, little is known about time lags and how they should be managed. This lack of knowledge puts those responsible for enabling translational research at a disadvantage. An ambitious reason for being able to accurately measure lags is that it would be possible to look at their distribution to identify research that is both slow and fast in its translation. Further investigation of the characteristics of research at both ends of a distribution could help identify actionable policy interventions that could speed up the translation process, where appropriate, and thus increase the return on research investment.

## DECLARATIONS

### Competing interests

None declared

### Funding

This is an independent paper funded by the Policy Research Programme in the Department of Health. The views expressed are not necessarily those of the Department

### Ethical approval

Not applicable

### Guarantor

JG

### Contributorship

ZSM designed, conducted and analysed the literature review, and drafted and revised the paper; JG initiated the project, drafted and revised the paper, and has led a number of studies cited that attempted to measure lags; SW revised the paper

### Acknowledgements

This paper derives from work undertaken by RAND Europe within Centre for Policy Research in Science and Medicine (PRISM), funded by the UK Department of Health. The authors thank NIHR for their support
